# Hazardous Alcohol Use and Its Effect on Direct-Acting Antiviral Therapy Initiation among People with Active Injection Drug Use and Current Hepatitis C Infection

**DOI:** 10.3390/v16091416

**Published:** 2024-09-05

**Authors:** Hamidreza Karimi-Sari, Gregory M. Lucas, Katie Zook, Brian Weir, Miles Landry, Susan G. Sherman, Kathleen R. Page, Oluwaseun Falade-Nwulia

**Affiliations:** 1 Division of Infectious Diseases, Johns Hopkins University School of Medicine, Baltimore, MD 21205, USA; hkarimi1@jhmi.edu (H.K.-S.); glucas@jhmi.edu (G.M.L.); kcook7@jhmi.edu (K.Z.); mlandry4@jhmi.edu (M.L.); kpage2@jhmi.edu (K.R.P.); 2 Department of Health, Behavior and Society, Johns Hopkins University Bloomberg School of Public Health, Baltimore, MD 21205, USA; bweir3@jhu.edu (B.W.); ssherman@jhu.edu (S.G.S.)

**Keywords:** hepatitis C virus, alcohol use disorder, intravenous drug use, antiviral agents

## Abstract

Background: Hepatitis C virus (HCV) infection and hazardous alcohol use are both preventable causes of morbidity and mortality among people who inject drugs (PWID). In the general population, hazardous alcohol is associated with a reduced likelihood of HCV treatment initiation. Less is known about the prevalence and impact of hazardous alcohol use on direct-acting antiviral (DAA) therapy initiation among PWID with active injection drug use. Methods: PWID were recruited via street outreach in Baltimore, Maryland, between 2018 and 2019 and were enrolled in a study cohort. Participants completed a study survey and underwent HCV testing. Self-reported DAA therapy initiation was evaluated at follow-up visits every six months. Hazardous alcohol use was determined based on an AUDIT-C score of ≥4 for men or ≥3 for women. Data were analyzed using multivariable logistic regression with generalized estimating equations. Results: Of the 720 PWID recruited, 291 had detectable HCV RNA, and only 134 were aware of their HCV infection. The mean (±standard deviation) age of those that were aware of their infection was 48.7 (±10.3) years, with a slight majority (53.0%) being male and predominantly African American (64.9%). The majority (80/134, 59.7%) met criteria for hazardous alcohol use. Only 16 (11.9%) PWID reported DAA therapy initiation within six months, and 20 (14.9%) reported it within 12 months of follow-up. Hazardous alcohol use (aOR = 1.23, 95% CI = 0.43–3.53) was not associated with DAA treatment initiation. Conclusions: There was a high prevalence of hazardous alcohol use, low rates of oral DAA therapy initiation, and no association between self-reported hazardous alcohol use and initiation of oral DAA therapy in our sample of PWID that were aware of their chronic HCV infection. Strategies to increase HCV treatment uptake in PWID with active drug use are urgently needed and should integrate alcohol and drug use evaluation and care.

## 1. Introduction

Hepatitis C virus (HCV) infection remains a significant public health issue that disproportionately impacts the health of people who use drugs [1,2]. Injection drug use stands out as a primary risk factor for HCV infection, with an estimated global HCV prevalence of 38.8% among people who inject drugs (PWID) [3], and 57% of new HCV infections in the United States (US) in 2020 attributed to injection drug use [4].

Alcohol use is associated with an increased risk of liver disease progression among people with HCV infection, accelerating progression to liver cirrhosis and hepatocellular cancer [5,6]. Alcohol use disorder is the most prevalent mental health disorder globally, with an estimated prevalence of 5.1%, and it is more prevalent among men (8.6%) compared to women (1.7%) [7]. According to the U.S. National Survey on Drug Use and Health, 21.7% of people aged 12 or older in the U.S. reported binge alcohol use in the past month. Additionally, the prevalence of alcohol use disorder in the past year (through 2019) was estimated to range from 4% to 9% among individuals aged 12 or older in different U.S. regions [8]. Concerns about lower adherence to HCV treatment [9,10] have resulted in a lower prescription rate of direct acting antiviral (DAA) therapy for individuals with alcohol use disorder [11]. In a study by Haque et al., alcohol use disorder was linked to a reduced likelihood of HCV treatment initiation among U.S. veterans with chronic HCV infection [12]. However, less is known about the prevalence of hazardous alcohol use and its impact on DAA therapy initiation among PWID with active drug use and current HCV infection. This study aimed to assess the prevalence and impact of hazardous alcohol use on HCV treatment initiation among individuals with active injection drug use in Baltimore, Maryland.

## 2. Materials and Methods

### 2.1. Study Participants

**Figure 1:**
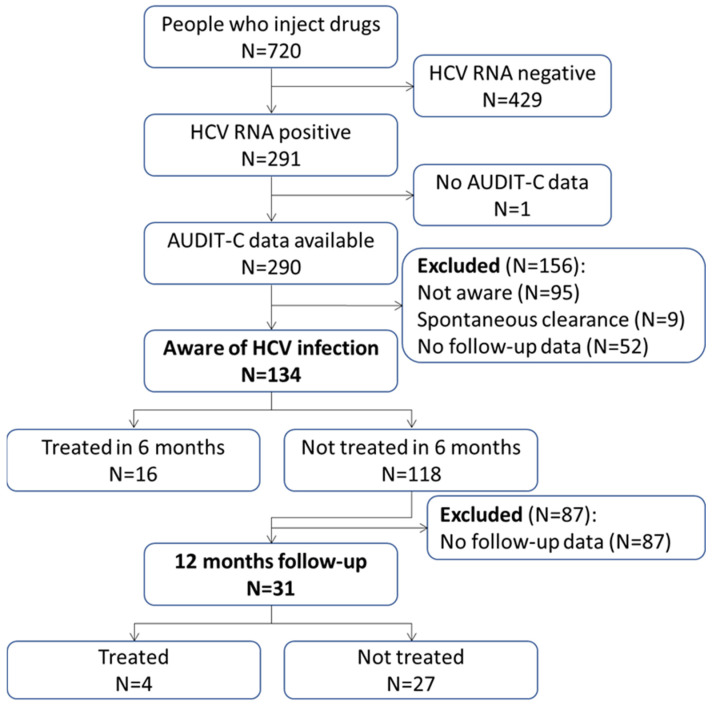
Study flowchart showing the number of participants included in the data analysis.

Data were collected from participants enrolled in the INSITE randomized clinical trial cohort (NCT03567174), the details of which have previously been described [13]. In brief, PWID were recruited via street outreach in Baltimore, Maryland, between 2018 and 2019 from 12 community settings adjacent to mobile syringe service program sites. Inclusion criteria included being 18 years of age or older, having a history of injection drug use, and either having a confirmed status of HIV infection or one of the following: engaging in injection drug use 4 or more days within the prior 30 days or reporting needle/syringe sharing within the prior 6 months. Exclusion criteria included incapacity to provide informed consent or being unwilling or unable to provide a blood sample. The INSITE study recruited 720 PWID across 12 sites (60 individuals per site). Our analysis focused on participants currently infected with HCV who were aware of their infection (Figure 1).

### 2.2. Data Collection

Participants completed a study survey at baseline, providing information on biological sex (male/female), level of formal education, race/ethnicity, employment status, insurance coverage, access to a primary care provider (PCP), and frequency of injection drug use (1, 2, 3, 4, or ≥5 times) per day further categorized as < or ≥3 times per day for analyses. Hazardous alcohol use was assessed using the validated Alcohol Use Disorders Identification Test—Concise (AUDIT-C) questionnaire, and was defined as an AUDIT-C score of ≥4 for men or ≥3 for women [14]. HCV antibody and RNA testing was performed on stored samples from all participants. Awareness of HCV infection status was assessed by asking participants if they had been diagnosed with HCV infection. Self-reported initiation of HCV treatment was evaluated during study follow-up at 6-month intervals.

### 2.3. Statistical Analysis

Analyses were performed in Stata statistical software (release 18, StataCorp LLC, College Station, TX, USA) for Windows. Characteristics of participants with and without hazardous alcohol use was assessed using Chi-squared (or Fisher’s exact) and t tests. McNemar’s test was performed to compare the rates of hazardous alcohol use between baseline and the 6-month follow-up. Univariable and multivariable logistic regression analyses with generalized estimating equations (GEEs) were utilized to evaluate the factors associated with HCV treatment initiation in 6-month intervals. A *p* value less than 0.05 was considered statistically significant.

### 2.4. Ethical Consideration

Participants provided written informed consent at enrollment. The study received approval from the Institutional Review Board of the Johns Hopkins University School of Medicine. Approval code: IRB00147873; approval date: December 2017.

## 3. Results

Among 720 PWID, 291 (40.4%) had current HCV infection, as evidenced by detectable HCV RNA, of which 290 provided AUDIT-C data. A total of 156 participants were excluded from subsequent analyses due to a lack of awareness of their HCV infection (95 participants), spontaneous clearance (9 participants), and lack of follow-up data (52 participants; Figure 1).

Among 134 people with HCV viremia and awareness of their HCV infection, the mean age (±standard deviation) was 48.7 ± 10.3 years, 53.0% were male, 64.9% were African American, and 60.4% reported >3 episodes of injection drug use per day.

Overall, 80 (60.4%) participants met the criteria for hazardous alcohol use. Participants with hazardous alcohol use were older than those without (51.3 ± 8.9 vs. 44.7 ± 11.1 years, *p* < 0.001). There were no significant differences by sex, education, race, employment, health insurance, having a PCP, HIV infection, and frequency of injection drug use between the participants with and without hazardous alcohol use (*p* > 0.05; Table 1). Among 290 participants with available AUDIT-C data, there was no significant difference in the rate of hazardous alcohol use between those included in the data analysis (134 participants) and those excluded (156 participants) (59.7% versus 51.3%, *p* = 0.151). The proportion of participants with hazardous alcohol use was 51.9% (27/52) among participants with no follow-up data, 52.6% (50/95) among those unaware of their HCV infection, and 33.3% (3/9) among participants with spontaneous HCV clearance. Compared to baseline, the proportion of participants with hazardous alcohol use decreased to 43.5% (57/131) at 6 months (*p* < 0.001). Notably, 29 participants with hazardous alcohol use at baseline no longer met the criteria at 6 months, while 9 participants without hazardous alcohol use at baseline met the criteria at 6 months.

DAA therapy was initiated in 16 (11.9%) PWID within 6 months of follow-up. Among 118 PWID who did not start HCV treatment within 6 months, 12 months follow-up data were available for 31 participants. In univariate analysis, DAA treatment was not significantly associated with hazardous alcohol use (OR = 1.84, 95% CI = 0.67–5.06), but was associated with age ≥50 years old (OR = 7.17, 95% CI = 2.04–25.26), male sex (OR = 3.13, 95% CI = 1.08–9.02), and Black race (OR = 4.48, 95% CI = 1.27–15.73). In multivariate analysis, hazardous alcohol use (aOR = 1.23, 95% CI = 0.43–3.53), male sex (aOR = 0.38, 95% CI = 0.13–1.11), Black race (aOR = 1.91, 95% CI = 0.63–5.83), and injection frequency (aOR = 0.93, 95% CI = 0.35–2.52) were not significantly associated with DAA treatment initiation. Older age ≥50 vs. <50 years was positively associated with HCV treatment initiation (aOR = 4.43, 95% CI = 1.51–13.02, Table 2).

## 4. Discussion

In our study of people with active injection drug use and current HCV infection, 60% of PWID met the criteria for hazardous alcohol use, putting them at an increased risk of liver disease progression from both alcohol and HCV infection [5,6]. This prevalence of hazardous alcohol use was much higher than that reported by others. In a community-recruited cohort of people with positive HCV antibodies and a history of injection drug use in Baltimore, 38% had hazardous alcohol use based on their AUDIT-C scores [15]. Similarly, in a prospective study conducted between 2002 and 2004 across Baltimore, MD; New York City, NY; and Seattle, WA, 37% of HCV-seropositive PWID reported problem drinking (AUDIT score ≥ 8) [16]. Notably, differing from these studies, all participants in our study were actively injecting drugs, which may explain the higher prevalence of hazardous alcohol use compared to studies including participants with a history of intravenous drug use. Other data have demonstrated an association between heroin/cocaine use and heavy levels of alcohol use [17,18]. Regardless of these differences, all these studies indicate high levels of alcohol use among people who use drugs, highlighting the need to screen and address alcohol use as a component of HCV care.

The lack of association between hazardous alcohol use and DAA therapy initiation in our sample of PWID with active drug use may be due to HCV treatment initiation in a low proportion of study participants within 6 months (11.9%) and 12 months (14.9%) after study enrolment. These data are consistent with other data demonstrating an association between recent (6 month) injection drug use and a lower likelihood of HCV treatment initiation [9,19]. In other populations with less injection drug use, alcohol use disorder has been recognized in the literature as a barrier to DAA prescription [11] and uptake [20]. In a study conducted among people with HCV infection recruited from a range of settings including hospital, community, and prison, individuals with alcohol contributing to liver disease were less likely to uptake HCV treatment [21]. Similarly, among people accessing care at four health care organizations in the United States between 2017 and 2018, individuals with hazardous alcohol use as evaluated by AUDIT-C were 42% less likely to initiate HCV treatment compared to those without hazardous alcohol use [19]. Other data among U.S. veterans have demonstrated an association between current alcohol use disorder and a lower likelihood of HCV treatment initiation compared to those with lower risk drinking [12]. However, the mentioned studies did not focus on people with active injection drug use.

Of note, in our sample of PWID, 33% (95 out of 290) were unaware of their infection, which is consistent with ongoing gaps in the US national HCV care continuum. Among 2.2 million U.S. adults with current HCV infection during 2017–2020, approximately 32.3% were unaware of their HCV infection [22].

Alcohol use contributes to cirrhosis [6], HCC [5], and higher mortality [23] in people with chronic HCV infection, even after HCV cure, putting PWID with hazardous alcohol use at an increased risk of negative HCV outcomes, which can be ameliorated through HCV treatment. Indeed, linkage to HCV care is an opportune time to evaluate for and address substance use including alcohol and other drug use. HCV treatment is recommended regardless of alcohol or drug use status [24]. Alcohol use evaluation, counseling, or treatment is also indicated as a component of HCV care [25]. HCV treatment may also alter subsequent alcohol use patterns. While several studies report a decrease in alcohol use after HCV treatment [26,27,28], others have found no change in alcohol use among people with HCV–HIV coinfection after DAA therapy [29].

Our findings are limited by the self-reported nature of alcohol use and DAA therapy initiation data and that the study population was limited to PWID in one urban city in Maryland and may not reflect the alcohol use patterns of PWID in other regions. The COVID-19 pandemic also limited study retention at 12 months, resulting in a lack of availability of data on all study participants at 12 months. Additionally, we assessed HCV treatment in the 6–12 months following notation of awareness of HCV infection and provision of linkage to care information. However, despite the short time period of observation for HCV treatment initiation, we believe the analysis of longitudinal data from PWID with active injection drug use is a significant strength of this study.

## 5. Conclusions

In our sample of PWID with active injection drug use, we found a high prevalence of hazardous alcohol use, and no association between self-reported hazardous alcohol use and the initiation of oral DAA therapy. HCV treatment initiation rates were overall very low. Strategies to increase linkage to care and uptake of HCV treatment, which also evaluate for and address alcohol and other drug use, are needed.

## Figures and Tables

**Table 1:** Participants’ characteristics among people with and without hazardous alcohol use ^1^.

Variable	Total (N = 134)	Hazardous Alcohol Use		*p* Value
		Yes (N = 80)	No (N = 54)	
Age, mean	48.7 ± 10.3	51.3 ± 8.9	44.7 ± 11.1	<0.001
Male, N (%)	71 (53.0)	46 (57.5)	25 (46.3)	0.202
GED or beyond, N (%)	53 (39.6)	32 (40.0)	21 (38.9)	0.897
African American race, N (%)	87 (64.9)	57 (71.2)	30 (55.5)	0.062
Hispanic, N (%)	5 (3.7)	3 (3.7)	2 (3.7)	0.989
Job, N (%)	9 (6.7)	6 (7.5)	3 (5.5)	0.659
Insurance, N (%)	122 (91.0)	74 (92.5)	48 (88.9)	0.473
PCP, N (%)	90 (67.2)	58 (72.5)	32 (59.3)	0.109
HIV infection, N (%)	24 (17.9)	16 (20.0)	8 (14.8)	0.443
Frequency of injections, N (%)				0.897
≤3 times a day	53 (39.6)	32 (40.0)	21 (38.9)	
>3 times a day	81 (60.4)	48 (60.0)	33 (61.1)	
Treatment initiation within 6 months, N (%)	16 (11.9)	10 (12.5)	6 (11.1)	0.808

^1^ General Educational Development (GED); primary care provider (PCP).

**Table 2:** Univariable and multivariable generalized estimating equations evaluating the factors associated with HCV treatment initiation within 12 months ^1^.

Variable	Odds Ratio (95% CI)	
	Unadjusted	Adjusted *
Hazardous alcohol use	1.84 (0.67–5.06)	1.23 (0.43–3.53)
Age ≥ 50	7.17 (2.04–25.26)	4.43 (1.51–13.02)
Male sex	3.13 (1.08–9.02)	2.66 (0.90–7.86)
African American race	4.48 (1.27–15.73)	1.91 (0.63–5.83)
>3 injections a day	0.54 (0.21–1.40)	0.93 (0.35–2.52)
GED or beyond	0.46 (0.16–1.35)	-
Job	0.71 (0.08–6.13)	-
PCP	3.36 (0.94–11.98)	-
HIV infection	0.85 (0.23–3.05)	-

^1^ 95% confidence interval (95% CI); * adjusted for hazardous alcohol use, age ≥50 years, male sex, African American race, and >3 injections per day.

## Data Availability

Data will be available upon request, as the dataset includes information on stigmatizing and illegal behaviors in combination with spatial information.
